# Intranasal IL-4 Administration Alleviates Functional Deficits of Periventricular Leukomalacia in Neonatal Mice

**DOI:** 10.3389/fneur.2020.00930

**Published:** 2020-09-02

**Authors:** Lin-chao Yu, Jing-kun Miao, Wei-bin Li, Na Chen, Qi-xiong Chen

**Affiliations:** ^1^Department of Neonatology, Children's Hospital of Chongqing Medical University, Chongqing, China; ^2^National Clinical Research Center for Child Health and Disorders, Chongqing, China; ^3^Ministry of Education Key Laboratory of Child Development and Disorders, Chongqing, China; ^4^Chongqing Key Laboratory of Pediatrics, Chongqing, China; ^5^Chongqing Key Laboratory of Child Health and Nutrition, Chongqing, China; ^6^Chongqing Health Center for Women and Children, Chongqing, China; ^7^Chongqing Hospital of Traditional Chinese Medicine, Chongqing, China

**Keywords:** IL-4 administration, intranasally, periventricular leukomalacia, remyelination, microglia

## Abstract

**Background:** Periventricular leukomalacia (PVL) is the major form of brain injury in premature infants. Currently, there are no therapies to treat PVL. Several studies suggested that polarization of microglia, a resident macrophage-like immune cell in the central nervous system, plays a vital role in brain injury and recovery. As an important mediator of immunity, interleukin-4 (IL-4) has critical effects on many immune cells, such as astrocytes and microglia. Increasing evidence shows that IL-4 plays a well-established role in attenuating inflammation in neurological disorders. Additionally, as a noninvasive and highly effective method, intranasal drug administration is gaining increasing attention. Therefore, in our study, we hypothesized that intranasal IL-4 administration is a promising strategy for PVL treatment.

**Methods:** The therapeutic effects of IL-4 on neuroprotection were evaluated using a Control group, Hypoxia group, and Hypoxia + IL-4 treatment group. The PVL mouse model was established by a severe acute hypoxia (SAH) protocol. Exogenous IL-4 was intranasally administered to investigate its neuroprotective effects. A functional study was used to investigate neurological deficits, immunohistochemical technology and Western blotting were used to detect protein levels, and electron microscopy was used to evaluate myelination.

**Results:** The results suggested that hypoxia stimulated Iba1^+^ microglial activation, downregulated myelin-related gene (*NG2, MAG*, and *MBP*) expression, reduced MBP protein levels, and caused neurological deficits. However, the intranasal administration of exogenous IL-4 partially inhibited Iba1+ microglial activation, improved myelination, and alleviated neurological deficits. The mechanistic study showed that IL-4 improved myelination possibly through the IL-4Ra-mediated polarization of microglia from the M1 phenotype to the M2 phenotype.

**Conclusion:** In summary, our findings demonstrated that the intranasal administration of exogenous IL-4 improves myelination and attenuates functional deficits in a hypoxia-induced PVL model. Intranasal IL-4 administration may be a promising strategy for PVL treatment, for which further mechanistic studies are urgent.

## Introduction

According to the WHO's estimates, births of very preterm infants (before 32 weeks of gestation) account for more than 2% of all live births, and the survival rates of these infants are more than 85% (1, 2), which is owing to advancements in obstetric and neonatal care (3). However, 25–50% of very preterm babies who survive still exhibit cognitive (4), visual (5), attention (3), and learning disabilities (6), such as cerebral palsy, which costs ~1 million dollars per person in the United States (7). Diffuse white matter injury is the most common form of injury in preterm birth infants, especially in infants with very low birth weights, and it is a condition that leads to periventricular leukomalacia (PVL) (8, 9). Many studies have found that PVL is associated with disruptions in the normal progression of preoligodendrocytes, which leads to prominent hypomyelination (10–12). Consistent with the effects of hypoxia–ischemia, preterm WMI is also accompanied by significant oxidative damage (13, 14). Microglial cells are resident macrophage-like cells in the central nervous system that have vital roles during brain development (15, 16). To participate in injury responses, immune regulation, and cytotoxic effects, microglia are capable of acquiring complex phenotypes (17). M1-polarized phenotypes are related to the secretion of nitrogen species and pro-inflammatory cytokines (18); M2-polarized phenotypes are associated with the secretion of growth factors and anti-inflammatory cytokines (18). Some studies have found that activated microglia, at least in the initial phase after injury, are involved in injury to immature white matter. In the acute phase, activated microglia, such as those with M1 phenotypes, have harmful effects on neurons and glia by releasing inflammatory cytokines, generating free radicals, and enhancing excitotoxicity (19, 20). However, after acute injury, some microglia, such as those with M2 phenotypes (M2a and M2c), might promote injury repair by being involved in late anti-inflammatory responses (21, 22).

Interleukin-4 (IL-4), a cytokine secreted primarily by Th2 cells, eosinophils, basophils, and mast cells, is a critical regulator of immunity (23, 24). Some studies have shown that IL-4 plays a central role in the production of the anti-inflammatory factors IL-10 and IL-13 while suppressing the generation of pro-inflammatory cytokines, such as IL-1, INF-a, and TNF-a (25, 26). In a focal ischemia model, the administration of exogenous IL-4 improved the neurological score, increased the spontaneous polarization, reduced the infarction volume, and decreased the infiltration of macrophages/microglia (27, 28), especially those with M2 phenotypes. However, loss of IL-4 increased the number of cells with the M1 phenotype (Iba1^+^iNOS^+^) and decreased the number of cells with the M2 phenotype (Iba1^+^Arg^+^) (28).

In our previous study, the IL-4 concentrations were significantly lower in asphyxiated newborn mice than in normoxic mice. Considering that IL-4 is associated with reduced macrophage/microglia infiltration and altered microglia phenotype ratios during hypoxia–ischemia injury to the brain, we tested the hypothesis that IL-4 reduces the release of pro-inflammatory cytokines, increases the production of anti-inflammatory factors, and finally reduces injury in a PVL mouse model.

## Methods and Materials

### Animals

Adult female C57BL/6 mice were purchased from Chongqing Medical University (Chongqing, China). The mice were housed under pathogen-free conditions. Water and food were available *ad libitum*. The female mice were crossed with age-matched male mice. Given that male preterm infants show more clinically relevant injuries and neurological impairments (29), only male pups were used. The mean anogenital distance from the caudal aspect of the genital area to the rostral aspect of the anus was used to assess neonatal gender (the mean distance is 1.9 ± 0.1 mm in males and 0.8 ± 0.1 mm in females) (30). The priori sample size was estimated by adequate statistical analysis. All the procedures were approved by the Experimental Animal Administration Committee of the university (followed the Chinese National Guidelines: GB/T 35892-20181) (31) and performed by qualified technicians following the 3R Principle: Reduction, Replacement, Refinement (32).

### Establishment of the PVL Model

All the male pups were randomly divided into three groups: the normoxia group (Control), the hypoxia-induced saline-treated group (Hypoxia), and the hypoxia-induced IL-4-treated group (Hypoxia + IL-4 group). The PVL animal model was established according to Clayton et al. and Shen et al. with modification (33, 34). Given the unstable state of C57BL/6 mice under hypoxic environments, all the male pups were fostered to gestational age-matched lactating CD1 (ICR) dams at postnatal day 1 (P1). Briefly, at P3, pups were randomly placed in a sealed chamber with the mother, and the O_2_ concentration was maintained at 7.5% by displacement with N_2_. Hypoxia began at P3 for 24 h; after 24 h, the hypoxic pups were returned to room air. The control pups breathed room air during the experiment. Half of the hypoxic pups were randomly selected to be subjected to IL-4 administration. After exposure, all the pups were returned to lactating CD1 (ICR) dams until sacrifice or weaning.

### Administration of Exogenous IL-4

IL-4 (mouse IL-4, 404-ML; R&D Systems) was prepared at a concentration of 100 ng μl^−1^ using a 0.45% normal saline solution and stored at −20°C. The pups in the Hypoxia group or the Hypoxia + IL-4 group were intranasally administered saline or IL-4 at a total dose of 80 ng g^−1^. The IL-4 treatment started 6 h after PVL and was repeated at postnatal days 5–7. Briefly, the mouse heads were fixed vertically, and IL-4 was administered by a microliter syringe (Hamilton CO., Reno, Nevada) into one nostril. After administration, the pups were held for 2 min to allow the IL-4 to be absorbed. The control animals were also administered the same volume of saline using the same procedure.

### Immunohistochemistry and Antibodies

At P4 and P11, mice were transcardially perfused with 4% paraformaldehyde, and white matter slices were sectioned with a microtome (Leica), as previously described (35, 36). Briefly, the slices were transferred to a slide, and fetal bovine serum was used to block the antigens. Then, the slices were incubated with primary antibodies for 12–16 h at 4°C; fluorescent-conjugated secondary antibodies were applied for 2–3 h at room temperature in the dark. To detect the maturity of oligodendrocytes, a human antibody against MBP was used. To detect the location of IL-4R, a rabbit antibody specific for phosphor-IL-4R was used. Microscopes (90I, Nikon, Japan) were used to detect the fluorescence values.

### Western Immunoblotting

The white matter was dissected, and the total protein was extracted with RIPA lysis buffer (Beyotime, Harman, China; RIPA: protease inhibitors =1,000:1), as previously described. The same protein amount (20 μg) was loaded onto sodium dodecyl sulfate-polyacrylamide gels and separated by electrophoresis (Bio-Rad, CA, USA). The proteins were transferred onto 0.2-μm PVDF membranes, and the membranes were incubated with different primary antibodies (see Table S1 for detail parameters) overnight at 4°C and with the appropriate secondary antibodies for 2 h at room temperature. An ECL kit (Millipore, USA) and an ECL Imaging System (Syngene G: BOX, UK) were used to detect protein expression. The images were analyzed using ImageJ software (NIH, Bethesda, Maryland).

### Real-Time PCR

The total RNA was extracted from the white matter of the mice at P4 and P7 according to the procedure of the RNA Extraction Kit (LS1040, Promega). Real-time PCR was performed using a CFX96 real-time PCR detection system (Bio-Rad) with SYBR reagents (QuantiNova SYBR® Green RT-PCR Kit, Qiagen). The relative expression of cDNA fragments was normalized using the average expression value of GAPDH and analyzed using the comparative C^T^ method (37). Real-time PCR was performed using the primer sequences shown in Table S2.

### ELISA

Whole brain tissues were collected at P7 to examine the concentration of IL-4 in the brain using a Mouse IL-4 ELISA kit [High Sensitivity; Neobioscience, EMC003(H)], according to the manufacturer's protocols. Briefly, the substrate was added to the plate and then placed directly in the spectrophotometer (462 nm wavelength, SpectraMax; Thermo, USA). The spectrophotometer and software (SoftMax, Release Pro 5) were programmed to shake the plate to homogenize the color in each well for 4 s before every reading, and the plate was read every 60 s until the end of the program. The sample levels were analyzed using a microtiter plate reader (Thermo, USA). Each sample was detected in duplicate, and the medians were used for analysis.

### Electron Microscopy

Mice at P18 were deeply anesthetized with pentobarbital sodium (50 mg/kg) and transcardially perfused with ice-cold phosphate-buffered solution containing 4.0% glutaraldehyde (pH 7.4). The brains were coronally sliced, and the white matter was dissected and postfixed for 2 weeks. All the samples were examined with an FEI transmission electron microscope (Tecnai G2 20 TWIN) and processed for standard electron microscopy analysis. Briefly, the white matter was dissected and postfixed for 2 weeks at room temperature (20°C). The samples were then dehydrated in 50–70–80–90–95–100–100% alcohol for 15 min each. The samples were incubated with a mixture solution (acetone:embedding medium = 1:1) overnight to permeabilize them, and then the samples were polymerized for 48 h at 60°C. A microtome (Leica UC7) was used to slice the tissue into 60- to 80-nm sections. Finally, uranium-lead (uranium-saturated aqueous solution of 2% acetate, lead citrate, 80096180) was used for staining for 15 min each. The slices were observed under a transmission electron microscope, and images were collected to calculate the percentage of unmyelinated axons [axons <0.3 μm were excluded (38)] and the *g* ratio, which was calculated as the axon diameter divided by the entire myelinated fiber (38, 39).

### FACS

Mice were deeply anesthetized and transcardially perfused with 100 ml of cold PBS, as described above. The M1 microglia and M2 microglia in the white matter were detected as previously described (40). The white matter in the mice in the Control, Hypoxia, and Hypoxia + IL-4 groups was harvested in FACS buffer (PBS with 1% FBS and 0.1% sodium azide) in a culture plate and homogenized with the rubber plunger of a 2-ml syringe on ice. Then, the cells were centrifuged at 3,000 rpm for 5 min at 4°C and resuspended in FACS buffer. Next, the cells were filtered through a 100-μm strainer, resuspended in 1 ml of FACS buffer and counted. The cells were stained with fluorochrome-labeled antibodies against CD45 (Biolegend, 368509, PE) and CD11b (Biolegend, 101211, APC) on ice for 40 min in the dark. The fluorescence threshold was set on the basis of the reactivity of the appropriate non-specific, fluorochrome-conjugated isotype controls. The data of the stained samples were acquired on a FACSCanto (a BD LSR II flow cytometer) using Diva software (v6.1.2; Institut Pasteur, Paris). The data were analyzed using FlowJo V10 software (v7.6.2; Tree Star, Ashland).

### Functional Study

Rotarod testing was conducted as previously described (41, 42). The functional test was conducted at P30 and P60 with the ANY-Maze Video Tracking System (ANY-Maze, USA). On the day before the test, the animals were brought to the testing room and allowed to rest for 2 h before testing. After a training session of two consecutive trials before the testing day, the mice were subjected to test sessions with two speed modes: accelerating speeds (range, from 0 to 50 rpm) and fixed speeds (fixed, 40 rpm). Each test mode consisted of two trials on the rotarod, with a maximum of 300 s. Each trial interval lasted more than 1 h. The time each mouse spent on the rotarod was recorded, and the average time of each trial was used for analysis (43).

### Statistics

In our study, 15–18 mice per group were analyzed in the functional study, and 4–6 mice per group were analyzed in the histochemical experiments. All the data are expressed as the mean ± S.E.M. and were analyzed by ANOVA (followed by Tukey's test) or unpaired Student's *t* test. GraphPad Prism (v.8.0.2) software and SPSS (v.20.0.0) software were used for all the statistical analyses, as appropriate. *P* < 0.05 was considered significant.

## Results

### Severe Acute Hypoxia Causes PVL in Neonatal Mice

**Figure 1:**
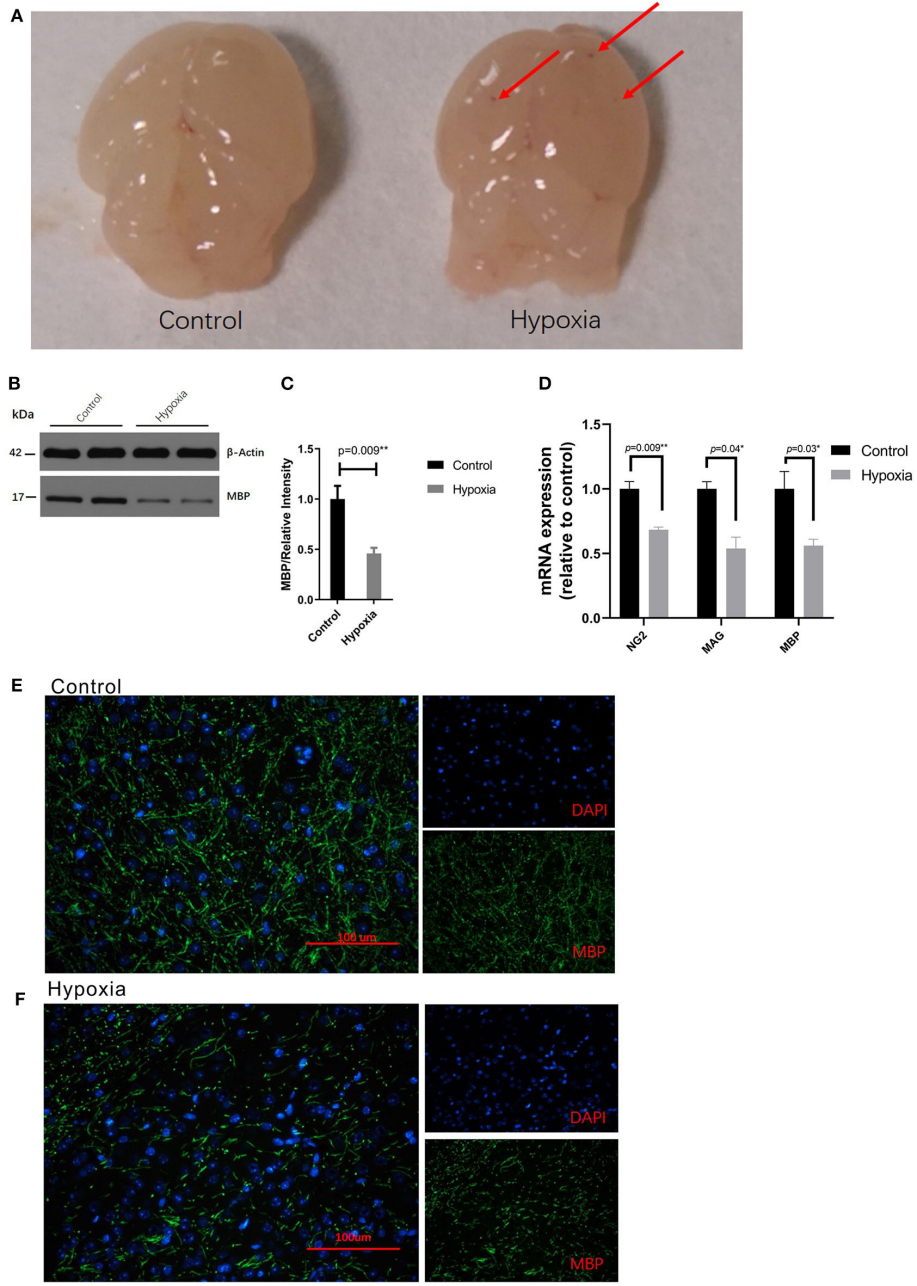
Severe acute hypoxia causes diffuse white matter injury. **(A)** Images of pathological brain specimens from the mice in the Control (left) and Hypoxia (right) groups at P4. Diffuse, small hemorrhagic spots (red arrow) were observed in brain tissue in the Hypoxia group but not in that in the Control group (*n* = 3 brains for each group). **(B)** Western immunoblotting of MBP in the white matter from P4 mice exposed to control or hypoxic conditions. The expression level of MBP (top panel) in the Hypoxia group was significantly lower than that in the Control group (*n* = 4 brains for each group, *p* = 0.01 by unpaired two-tailed *t* test). The histograms represent the normalized relative quantitative values between the Control group and Hypoxia group. **(C)** Normalized relative quantitative values of the immunohistochemistry analysis of MBP between the Control group and Hypoxia group. **(D)** Gene expression analysis of myelin-related mRNA (Ng2, Mag, and Mbp) in the white matter of P4 mice exposed to control or hypoxic conditions. The gene expression levels of Ng2, Mag, and Mbp in the Hypoxia group were significantly downregulated compared to those in the Control group (*n* = 4–5 brains for each group, *p* = 0.09, 0.04, and 0.03, respectively, by unpaired two-tailed *t* test). GAPDH was used to normalize the gene expression levels. **(E,F)** Immunohistochemistry analysis of MBP in the white matter from the P4 mice exposed to control **(E)** or hypoxic **(F)** conditions. Reduced MBP (green) was shown in the Hypoxia group compared to that in the Control group. The exposure time was 50 ms in all the scopes (*n* = 4–5 brain per group, *p* = 0.01 by unpaired two-tailed *t* test). All the data are expressed as the mean ± S.E.M. MBP, myelin basic protein; Ng2, nerve-glia antigen 2; Mag, myelin-associated glycoprotein; Mbp, myelin basic protein.

We established the preterm PVL model by placing mice in severe acute hypoxia or room air conditions, according to the methods described by Clayton et al. with brief modification (33). It was observed that reduced myelination due to a disrupted oligodendrocyte progenitor (preOL) pool contributes to PVL. Based on the images of the pathological brain specimens, diffuse, small hemorrhagic spots were clearly observed at the tissue surface at P4 in the Hypoxia group but were not observed at that in the Control group (Figure 1A). Regarding protein expression, hypoxia dramatically decreased the expression of myelin basic protein (MBP) in the white matter area of P4 mice compared to that in the white matter area of Control mice as determined by Western blotting (Figure 1B) and immunohistochemistry (Figures 1C,E,F). The expression of myelin-related mRNA (*Ng2, Mag*, and *Mbp*) in the white matter of hypoxic mice at P4 was obviously downregulated compared with that of the control mice (Figure 1D). These data showed that the PVL model had been successfully established.

### Administration of Exogenous IL-4 Alleviates Functional Deficits in PVL

**Figure 2:**
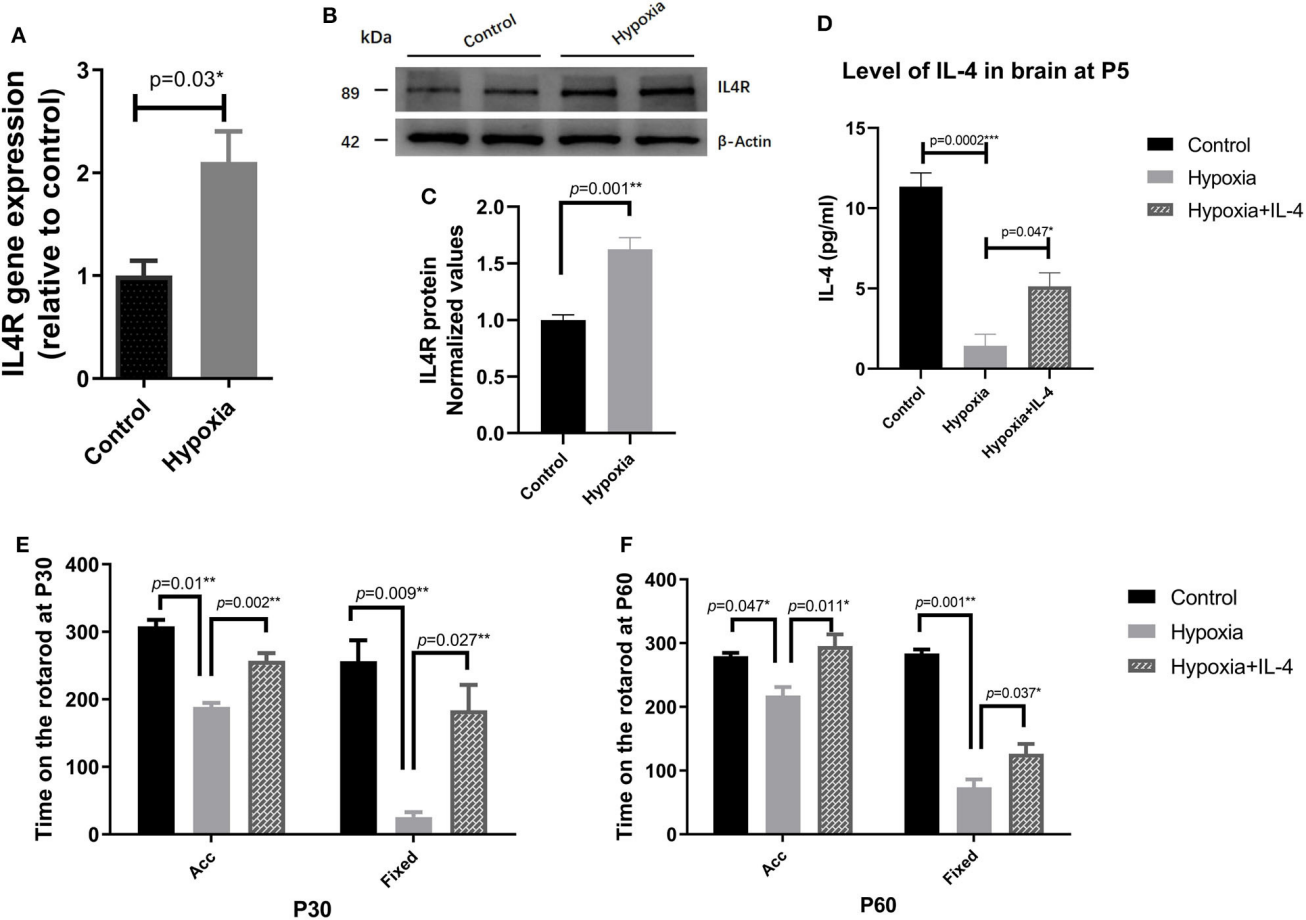
The administration of exogenous IL-4 improves the functional deficits of PVL. **(A)** Gene expression analysis of the IL-4 receptor in the white matter of the P4 mice exposed to hypoxia or control conditions. The mRNA levels of the IL-4 receptor were significantly higher in the Hypoxia group than in the Control group (*n* = 4–5 mice per group, *p* = 0.008 by ANOVA). **(B)** Western immunoblotting of IL-4R in the white matter of the P4 mice exposed to control or hypoxic conditions. The expression level of IL-4R (top panel) in the Hypoxia group was significantly higher than that in the Control group (*n* = 5 brains for each group, *p* = 0.01 by unpaired two-tailed *t* test). **(C)** The histograms represent the normalized relative quantitative values between the Control group and Hypoxia group. **(D)** Concentration of IL-4 in the P7 brains of the mice in the Control, Hypoxia, and Hypoxia + IL-4 groups (*n* = 6–7 mice per group, *p* = 0.0002 and 0.047, respectively, by ANOVA). **(E,F)**, Rotarod test of the mice in the Control, Hypoxia, and Hypoxia + IL-4 groups at P30 **(E)** and P60 **(F)**. The mice in the Hypoxia group exhibited shorter times on the rotarod than those in the Control group in different modes at P30 and P60 (*p* = 0.01, 0.009; 0.047, 0.001, respectively). However, the administration of IL-4 partially rescued the time on the rotarod compared to the administration of saline to the hypoxia mice in different modes at P30 and P60 (*n* = 15–18 mice per group, *p* = 0.002, 0.027; 0.011, 0.037 by ANOVA). Acc, acceleration pattern; Fixed, fixed pattern.

It is widely reported that IL-4 is closely associated with many brain injury diseases, such as cerebral infarction (44), transient focal cerebral ischemia (27, 28), neonatal asphyxia (45), and hypoxic–ischemic encephalopathy (46). First, we performed a dose-response experiment at the beginning of our research. We set the dose of IL-4 to be intranasally administered to a total of 0, 40, 80, and 120 ng g^−1^, and functional assessments were conducted at P30, as shown in Figure S2. Functional differences were significantly detected in the mice administered 80 and 120 ng g^−1^ IL-4. Therefore, we reported the concentration of 80 ng g^−1^ in the study. To explore the effect of IL-4 on PVL in a mouse model, the concentration of IL-4 in the brain tissue and the expression of IL-4R in the white matter of the mice exposed to control and hypoxia conditions at P4 were detected. Obviously, decreased levels of IL-4 in the brain tissue and increased expression of IL-4R in the white matter were found in the mice exposed to hypoxia compared to those in the mice exposed to control conditions (Figures 2A–D). Exogenous IL-4 was intranasally administered to easily penetrate through the blood–brain barrier. To evaluate motor coordination and learning, rotarod testing was conducted with the mice in the Control, Hypoxia, and Hypoxia + IL-4 groups. At P30, the functional study showed that the hypoxic mice spent less time on the rotarod than the control mice. However, the administration of exogenous IL-4 caused the hypoxic mice to spend obviously more time on the rotarod than the mice exposed to hypoxia and administered saline (Figure 2E). Comparable results were observed in the mice at P60 (Figure 2F).

### Administration of Exogenous IL-4 Improves Myelination

**Figure 3:**
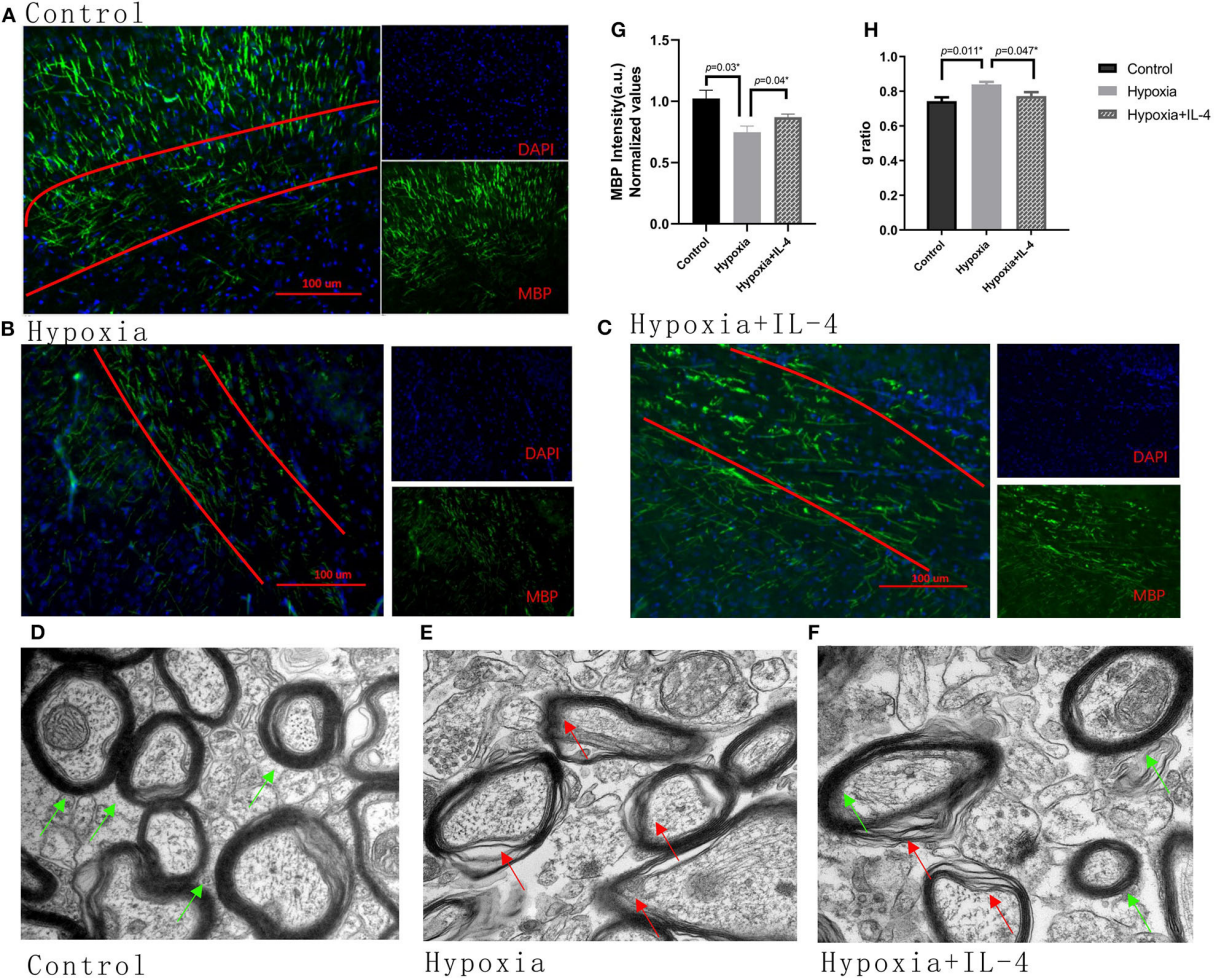
Administration of exogenous IL-4 improves myelination. **(A–C)** Immunohistochemistry analysis of MBP at P11 in the white matter of the Control, Hypoxia, and Hypoxia + IL-4 groups. The level of MBP in the mice exposed to hypoxia **(B)** was significantly reduced compared with that in the mice exposed to control conditions **(A)** (*n* = 4–5 brains per group, *p* = 0.03 by ANOVA). However, the administration of exogenous IL-4 to the mice exposed to hypoxia **(C)** partly rescued myelination compared to the administration of saline to the mice exposed to hypoxia (*n* = 4–6 brains per group, *p* = 0.04 by ANOVA). The exposure time was 50 ms in all the scopes. **(G)** Normalized relative quantitative values of the immunohistochemistry analysis of MBP among the Control group, Hypoxia group, and Hypoxia + IL-4 group. **(D–F)** Electron microscopy images of the axons in the white matter of the P11 mice in the Control **(D)**, Hypoxia **(E)**, and Hypoxia + IL-4 **(F)** groups. **(H)** *g* ratio of axon myelination. There was an increased *g* ratio in the Hypoxia group compared with that in the Control group (*n* = 3 brains per group, more than 20 axons were calculated for each brain, *p* = 0.011 by ANOVA). However, the administration of IL-4 to the mice exposed to hypoxia decreased the *g* ratio compared to the administration of saline to the mice exposed to hypoxia (*p* = 0.047 by ANOVA).

The improvements observed in the functional study urged us to examine the recovery of myelination in the mouse brain. The expression of MBP in the brain was measured by immunohistochemistry at P11 and by electron microscopy at P18. The mice exposed to hypoxia exhibited obviously delayed myelination in the brain compared to the mice exposed to control conditions. Interestingly, the intranasal administration of exogenous IL-4 to the mice exposed to hypoxia rescued myelination compared with the administration of saline to the hypoxic mice (Figures 3A–C,G). The *g* ratio (calculated as the axon diameter divided by the entire myelinated fiber) was used to assess the thickness of the myelin sheath. The microscopy results showed that the hypoxic mice had thinner myelination and a higher *g* ratio than the control mice. However, exogenous IL-4 administration to the hypoxic mice improved myelination and reduced the *g* ratio compared with the administration of saline to the hypoxic mice (Figures 3D–F,H). These data suggested that the intranasal administration of exogenous IL-4 rescued the expression of MBP and improved myelination.

### Administration of Exogenous IL-4 Increases the Level of IL-4 in the Brain

To explore the role of IL-4 in the PVL model, the concentration of IL-4 in the brain at P7 was measured by ELISA. The level of IL-4 in the brains of mice exposed to hypoxia was lower than that in the brains of mice exposed to control conditions (Figure 2D). The intranasal administration of exogenous IL-4 increased the level of IL-4 in the hypoxic mouse brain (Figure 2D), indicating that the intranasal administration of IL-4 increased the concentration of IL-4 in the lesion area.

### Regulating the Effect of IL-4, Which Is Partially Dependent on Microglia Polarization

**Figure 4:**
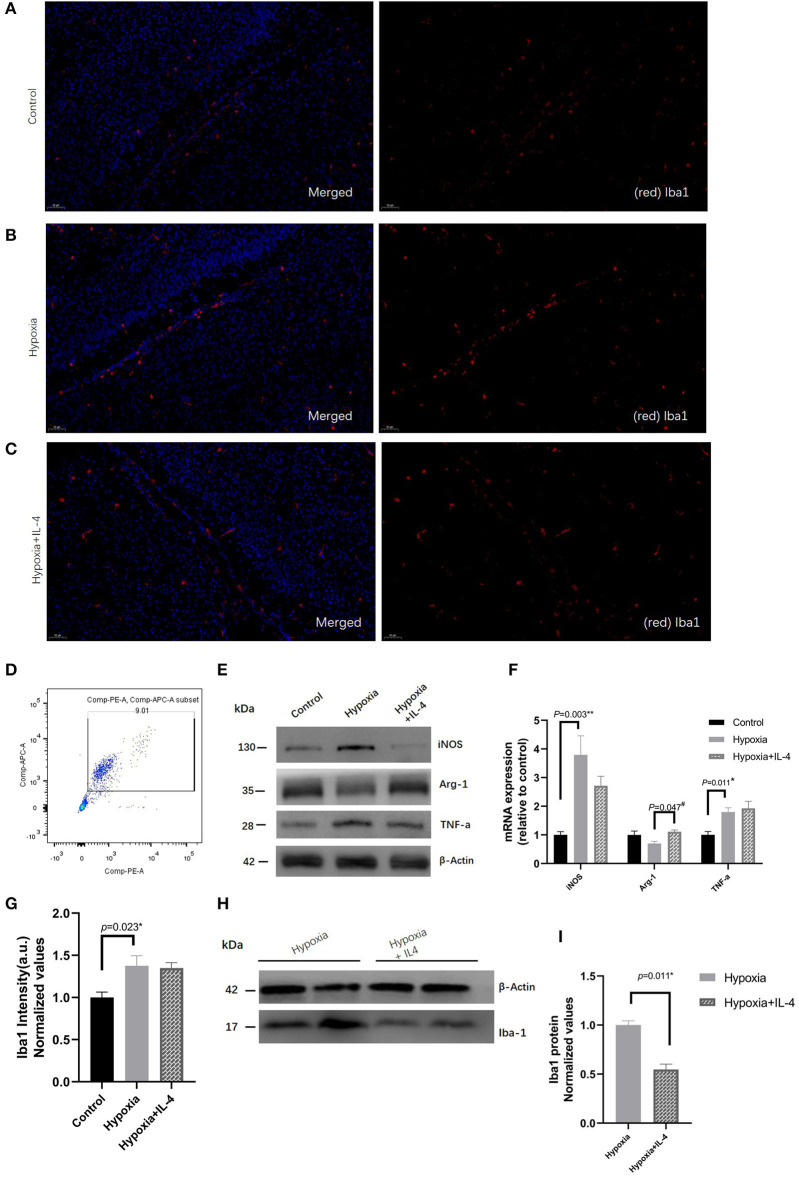
Immunohistochemistry analysis of Iba1 in the white matter of P7 mice in the Control, Hypoxia, and Hypoxia + IL-4 groups. **(A–C)** Iba1+ cell staining at P7 in the white matter of the Control, Hypoxia, and Hypoxia + IL-4 groups. The numbers of microglia cells in the mice exposed to hypoxia **(B)** were significantly increased compared with those in the mice exposed to control conditions **(A)** (*n* = 4–5 brains per group, *p* = 0.023 by ANOVA). Additionally, the administration of exogenous IL-4 to mice exposed to hypoxia **(C)** slightly alleviated the activation of microglia compared to the administration of saline to the mice exposed to hypoxia (*n* = 4–5 brains per group, no significant difference, ANOVA). The exposure time was 50 ms in all the scopes. **(G)** Normalized relative quantitative values of immunohistochemistry analysis of Iba1 among the Control group **(A)**, Hypoxia group **(B)**, and Hypoxia + IL-4 **(C)** group. **(E)** mRNA expression of iNOS, Arg1, and TNF-a in the microglial cells sorted by FACS at P7 from the white matter of the mice in the Control, Hypoxia, and Hypoxia + IL-4 groups. **(F)** Western immunoblotting of iNOS, Arg-1, and TNF-a in the microglial cells sorted by FACS at P7 from the white matter of the mice in the Control, Hypoxia, and Hypoxia + IL-4 groups. Increased iNOS and TNF-a expression levels and decreased Arg-1 expression levels were observed in the Hypoxia group compared with the Control group. However, IL-4 administration reversed the expression of these proteins (*n* = 4 brains for each group). **(D)** Microglial cells sorted by FACS. **(H)** Western immunoblotting of Iba1 at P7 in the white matter of the mice in the Hypoxia and Hypoxia + IL-4 groups. The expression level of Iba1 in the Hypoxia + IL-4 group was significantly lower than that in the Hypoxia group (*n* = 4 brains for each group, *p* = 0.011 by unpaired two-tailed *t* test). **(I)** Normalized relative quantitative values of Western immunoblotting analysis of Iba1 in the P7 mice between the Hypoxia group and Hypoxia + IL-4 group. FACS, fluorescence-activated cell sorting.

Many studies have found that microglia are involved in the neuroinflammation that protects against brain damage. To explore the role of microglial polarization in hypoxia, the microglia in the white matter zone were quantitatively analyzed by immunohistochemistry and Western blotting experiments. The quantitative results of the microglia in the white matter showed that exposure to hypoxia increased the numbers of Iba^+^ microglia (Figures 4A–C,G–I). Interestingly, IL-4 administration slightly decreased the numbers of Iba^+^ microglia (Figures 4A–C,G). In addition, microglial cells (CD45^+^CD11b^+^) were sorted by FACS (Figure 4D), and the gene and protein expression of iNOS, Arg1, and TNF-a were detected. Decreased iNOS and TNF-a expression and increased Arg1 expression were observed in the mice in the Hypoxia + IL-4 group compared with those in the Hypoxia group (Figures 4E,F), suggesting that the administration of IL-4 promotes microglial polarization from the M1 phenotype (CD45^+^CD11b^+^, high *iNOS* expression) to the M2 phenotype (CD45^+^CD11b^+^, high *Arg1* expression).

## Discussion

As the most common form of injury in infants with very low birth weights, PVL represents a large burden to the health system (7–9). Previous studies have revealed the potent neuroprotective effects of IL-4 in animal models (27, 28). Myelination is considered to be correlated with the manifestation of PVL (10, 11). Therefore, we performed the present study using the hypoxia model to investigate the effect of IL-4 in PVL. In the present study, we found that the intranasal administration of exogenous IL-4 improved the myelination of axons, and the improvement of myelination might partly depend on microglial polarization in the brain. These results show that IL-4 is important in modulating the improvement of myelination in PVL.

As the most potent immune cells in the CNS, microglial cells play an important role in CNS repair and regeneration (47, 48). Disruptions of myelination in early stages have deleterious consequences for the development of neuronal connectivity, such as in PVL, in both animal models and human specimens (13). The polarized phenotypes of microglial cells have distinct roles in the injury and recovery of white matter. Consistent with previous studies, our results suggested that hypoxia obviously increased the polarization of microglia (increased Iba1+ cell activation, increased iNOS expression, and decreased Arg1 expression) and caused a reduction in myelination (decreased MBP expression) in the mouse PVL model. In an *in vitro* model of inflammation, Chhor V et al. proved that stimulation with anti-inflammatory cytokines increased the expression of genes associated with the M2 microglial phenotype (such as Arg1); however, the expression of genes associated with the M1 phenotype (such as iNOS) was increased by stimulation with pro-inflammatory mediators (17). In our study, the gene and protein expression of iNOS, Arg1, and TNF-a were measured in FACS-sorted microglia by RT-PCR and Western blotting. In accordance with reported studies, our findings showed increased iNOS expression levels in CD45^+^CD11b^+^ cells and decreased Arg1 expression levels in CD45^+^CD11b^+^ cells. These results showed that microglial cells are closely related to myelination disturbances in the PVL mouse model.

As a T cell-derived mediator, IL-4 plays a critical role in several neurological disorders (49). Derecki N et al. reported that IL-4^−/−^ mice exhibited severe learning abnormalities compared with wild-type mice. In addition, increased production of IL-4 was detected in mice training in the Morris water maze compared to that in untrained mice (50). In a focal ischemia mouse model, Xiong X et al. showed that IL-4 KO mice exhibited larger infarction volumes and worsened neurological scores than wild-type mice. In our study, our results showed that the administration of exogenous IL-4 improved myelination and reduced the polarization of microglia to the M1 phenotype. Furthermore, IL-4 alleviated the hypoxia-induced functional impairment in a PVL mouse model. As an immune cytokine, IL-4 has been reported to regulate the expression of MHC class II molecules and the enhancement of macrophage mannose receptor activity (51). In addition, Chhor V et al. found that stimulation with IL-4 increased the gene expression of M2 markers (such as Arg-1 and CD206) but did not increase the expression of M1 markers (such as TNF-a, IL-6, and IL-1b). Similarly, our results showed that the administration of exogenous IL-4 decreased iNOS expression in CD45^+^CD11b^+^ cells and increased Arg1 expression in CD45^+^CD11b^+^ cells. These results showed that IL-4 attenuates myelination disturbances and functional impairments by regulating microglia polarization in a PVL mouse model.

However, despite an increasing body of evidence supporting the beneficial effects of IL-4 in neurological diseases, the effect of IL-4 in hypoxia-induced brain injuries is under debate (44). Kim et al. reported that IL-4 levels were increased in patients with cerebral infarction (44). However, our results showed reduced IL-4 levels in the hypoxia-induced model. Many possibilities may explain this controversy. (1) Different tissues (serum and brain) exhibit different cytokine levels. Due to interference by the blood–brain barrier, the IL-4 levels in the brain may differ from those in serum. (2) Different experimental models (cerebral infarction and PVL models, human, and mouse models) may generate different IL-4 levels. The IL-4 receptor alpha chain (IL-4Ra) has been reported to mediate the effects of IL-4 signaling. When IL-4Ra binds its ligand, IL-4Ra can dimerize with the gamma chain or the IL-13 receptor alpha1, producing a type 1 complex or type 2 complex, respectively (52). In our results, we found increased IL-4Ra protein levels and decreased IL-4 concentrations in the PVL-induced mouse model. Part of the reason for these observations is probably because of compensatory mechanisms. A reduced ligand level would promote an increase in ligand receptors to reduce changes in the downstream signaling pathways. The mechanism of cerebral infarction in patients is obviously different from that in the PVL mouse model. However, the different effects observed in PVL models and infarction models remind us that IL-4 might play distinct roles in different diseases. In addition, our research only showed the correlation between the administration of exogenous IL-4 and the alleviation of functional deficits, and we did not include IL-4^−/−^ transgenic mice in our study. Our team will address this limitation in our future work. Overall, intranasal IL-4 administration improved myelination and alleviated functional deficits, as presented in this study, which might be explained by the polarization of microglia, including the regulation of *iNOS* and *Arg1* expression in CD45^+^CD11b^+^ cells. These results might suggest the possibility of treating PVL by targeting the polarization of microglia.

## Conclusion

Overall, our findings demonstrated that the intranasal administration of exogenous IL-4, at least in a hypoxia-induced PVL model, could improve myelination and attenuate functional deficits. These results show that intranasal IL-4 administration may be a new strategy for PVL treatment. Polarization of microglia is an important but not exclusive mechanism and needs to be further explored.

## Data Availability Statement

All datasets generated for this study are included in the article/Supplementary Material.

## Ethics Statement

The animal study was reviewed and approved by the Experimental Animal Administration Committee of the University.

## Author Contributions

LY and QC were responsible for study concept and collated data and prepared manuscript. LY, NC, and JM carried out the studies and analyzed data. WL were responsible for working draft and ethical requirements concerning animal welfare. All authors contributed to the article and approved the submitted version.

## Conflict of Interest

The authors declare that the research was conducted in the absence of any commercial or financial relationships that could be construed as a potential conflict of interest.

## References

[1] Als H Behrman R Checchia P Denne S Dennery P Hall CB. Preemie abandonment? Multidisciplinary experts consider how to best meet preemies needs at “preterm infants: a collaborative approach to specialized care” roundtable. Modern Healthcare. (2007) 37:17–24. 17607906

[2] Horbar JD Badger GJ Carpenter JH Fanaroff AA Kilpatrick S LaCorte M. Trends in mortality and morbidity for very low birth weight infants, 1991–1999. Pediatrics. (2002) 110(1 Pt 1):143–51. 10.1542/peds.110.1.143 12093960

[3] Anderson PJ De Luca CR Hutchinson E Spencer-Smith MM Roberts G Doyle LW. Attention problems in a representative sample of extremely preterm/extremely low birth weight children. Dev Neuropsychol. (2011) 36:57–73. 10.1080/87565641.2011.540538 21253991

[4] Soria-Pastor S Padilla N Zubiaurre-Elorza L Ibarretxe-Bilbao N Botet F Costas-Moragas C. Decreased regional brain volume and cognitive impairment in preterm children at low risk. Pediatrics. (2009) 124:e1161–70. 10.1542/peds.2009-0244 19948618

[5] Glass HC Fujimoto S Ceppi-Cozzio C Bartha AI Vigneron DB Barkovich AJ. White-matter injury is associated with impaired gaze in premature infants. Pediatr Neurol. (2008) 38:10–5. 10.1016/j.pediatrneurol.2007.08.019 18054686 PMC2203614

[6] Litt J Taylor HG Klein N Hack M. Learning disabilities in children with very low birthweight: prevalence, neuropsychological correlates, and educational interventions. J Learn Disabil. (2005) 38:130–41. 10.1177/00222194050380020301 15813595

[7] CfDCaP C. Economic costs associated with mental retardation, cerebral palsy, hearing loss and vision impairment–United States, 2003. MMWR Morb Mortal Wkly Rep. (2003) 53:7–9. 14749614

[8] Loeliger M Inder T Cain S Ramesh RC Camm E Thomson MA. Cerebral outcomes in a preterm baboon model of early versus delayed nasal continuous positive airway pressure. Pediatrics. (2006) 118:1640–53. 10.1542/peds.2006-0653 17015557

[9] Buser JR Maire J Riddle A Gong X Nguyen T Nelson K. Arrested preoligodendrocyte maturation contributes to myelination failure in premature infants. Anna Neurol. (2012) 71:93–109. 10.1002/ana.22627 22275256 PMC3270934

[10] Volpe JJ. Brain injury in premature infants: a complex amalgam of destructive and developmental disturbances. Lancet Neurol. (2009) 8:110–24. 10.1016/S1474-4422(08)70294-1 19081519 PMC2707149

[11] Haynes RL Billiards SS Borenstein NS Volpe JJ Kinney HC. Diffuse axonal injury in periventricular leukomalacia as determined by apoptotic marker fractin. Pediatr Res. (2008) 63:656–61. 10.1203/PDR.0b013e31816c825c 18520330 PMC2770332

[12] Riddle A Maire J Gong X Chen KX Kroenke CD Hohimer AR. Differential susceptibility to axonopathy in necrotic and non-necrotic perinatal white matter injury. Stroke. (2012) 43:178–84. 10.1161/STROKEAHA.111.632265 22076007 PMC3246543

[13] Back SA. White matter injury in the preterm infant: pathology and mechanisms. Acta Neuropathol. (2017) 134:331–49. 10.1007/s00401-017-1718-6 28534077 PMC5973818

[14] Back SA Luo NL Mallinson RA O'Malley JP Wallen LD Frei B. Selective vulnerability of preterm white matter to oxidative damage defined by F2-isoprostanes. Annals Neurol. (2005) 58:108–20. 10.1002/ana.20530 15984031

[15] Giulian D Baker TJ. Characterization of ameboid microglia isolated from developing mammalian brain. J Neurosci. (1986) 6:2163–78. 10.1523/JNEUROSCI.06-08-02163.1986 3018187 PMC6568755

[16] Glenn JA Ward SA Stone CR Booth PL Thomas WE. Characterisation of ramified microglial cells: detailed morphology, morphological plasticity and proliferative capability. J Anatomy. (1992) 180(Pt 1):109–18. 1452465 PMC1259614

[17] Chhor V Le Charpentier T Lebon S Ore MV Celador IL Josserand J. Characterization of phenotype markers and neuronotoxic potential of polarised primary microglia *in vitro*. Brain Behav Immun. (2013) 32:70–85. 10.1016/j.bbi.2013.02.005 23454862 PMC3694309

[18] Edwards JP Zhang X Frauwirth KA Mosser DM. Biochemical and functional characterization of three activated macrophage populations. J Leukocyte Biol. (2006) 80:1298–307. 10.1189/jlb.0406249 16905575 PMC2642590

[19] Lehnardt S Lachance C Patrizi S Lefebvre S Follett PL Jensen FE. The toll-like receptor TLR4 is necessary for lipopolysaccharide-induced oligodendrocyte injury in the CNS. J Neurosci. (2002) 22:2478–86. 10.1523/JNEUROSCI.22-07-02478.2002 11923412 PMC6758325

[20] Lehnardt S Massillon L Follett P Jensen FE Ratan R Rosenberg PA. Activation of innate immunity in the CNS triggers neurodegeneration through a Toll-like receptor 4-dependent pathway. Proc Natl Acad Sci USA. (2003) 100:8514–9. 10.1073/pnas.1432609100 12824464 PMC166260

[21] Butovsky O Landa G Kunis G Ziv Y Avidan H Greenberg N. Induction and blockage of oligodendrogenesis by differently activated microglia in an animal model of multiple sclerosis. J Clin Invest. (2006) 116:905–15. 10.1172/JCI26836 16557302 PMC1409740

[22] Hagberg H Mallard C Ferriero DM Vannucci SJ Levison SW Vexler ZS. The role of inflammation in perinatal brain injury. Nat Rev Neurol. (2015) 11:192–208. 10.1038/nrneurol.2015.13 25686754 PMC4664161

[23] Howard M Farrar J Hilfiker M Johnson B Takatsu K Hamaoka T. Identification of a T cell-derived b cell growth factor distinct from interleukin 2. J Exp Med. (1982) 155:914–23. 10.1084/jem.155.3.914 6977612 PMC2186613

[24] Hu-Li J Shevach EM Mizuguchi J Ohara J Mosmann T Paul WE. B cell stimulatory factor 1. (interleukin 4) is a potent costimulant for normal resting T lymphocytes. J Exp Med. (1987) 165:157–72. 10.1084/jem.165.1.157 3098893 PMC2188254

[25] Hart PH Vitti GF Burgess DR Whitty GA Piccoli DS Hamilton JA. Potential antiinflammatory effects of interleukin 4: suppression of human monocyte tumor necrosis factor alpha, interleukin 1, and prostaglandin E2. Proc Natl Acad Sci USA. (1989) 86:3803–7. 10.1073/pnas.86.10.3803 2786204 PMC287229

[26] Abbas AK Murphy KM Sher A. Functional diversity of helper T lymphocytes. Nature. (1996) 383:787–93. 10.1038/383787a0 8893001

[27] Xiong X Barreto GE Xu L Ouyang YB Xie X Giffard RG. Increased brain injury and worsened neurological outcome in interleukin-4 knockout mice after transient focal cerebral ischemia. Stroke. (2011) 42:2026–32. 10.1161/STROKEAHA.110.593772 21597016 PMC3128567

[28] Xiong X Xu L Wei L White RE Ouyang YB Giffard RG. IL-4 Is required for sex differences in vulnerability to focal ischemia in mice. Stroke. (2015) 46:2271–6. 10.1161/STROKEAHA.115.008897 26130091 PMC4519392

[29] Ment LR Vohr B Allan W Katz KH Schneider KC Westerveld M. Change in cognitive function over time in very low-birth-weight infants. JAMA. (2003) 289:705–11. 10.1001/jama.289.6.705 12585948

[30] Schneider J Nyby J Whitney G Determining the sex of neonatal mice. (Mus musculus). Behav Res Methods Instrum. (1978) 10:105 10.3758/BF03205110

[31] MacArthur Clark JA Sun D. Guidelines for the ethical review of laboratory animal welfare People's Republic of China National Standard GB/T 35892–2018 [Issued 6 February 2018 Effective from 1 September 2018]. Anim Models Exp Med. (2020) 3:103–13. 10.1002/ame2.12111 32318667 PMC7167230

[32] Lu J Bayne K Wang J. Current status of animal welfare and animal rights in China. Alternat Lab Animals. (2013) 41:351–7. 10.1177/026119291304100505 24329743

[33] Clayton BL Huang A Kunjamma RB Solanki A Popko B. The integrated stress response in hypoxia-induced diffuse white matter injury. J Neurosci. (2017) 10.1523/JNEUROSCI.2738-16.2017 28720571 PMC5546113

[34] Shen Y Plane JM Deng W. Mouse models of periventricular leukomalacia. J Visualized Exp. (2010). 39:2738–16. 10.3791/1951 20485263 PMC3149994

[35] Fritschy JM. Is my antibody-staining specific? How to deal with pitfalls of immunohistochemistry. Eur J Neurosci. (2008) 28:2365–70. 10.1111/j.1460-9568.2008.06552.x 19087167

[36] Liu HJ Lai X Xu Y Miao JK Li C Liu JY. alpha-asarone attenuates cognitive deficit in a pilocarpine-induced status epilepticus rat model via a decrease in the nuclear factor-kappab activation and reduction in microglia neuroinflammation. Front Neurol. (2017) 8:661. 10.3389/fneur.2017.00661 29312110 PMC5735142

[37] Gao F Yang YZ Feng XY Fan TT Jiang L Guo R. Interleukin-27 is elevated in sepsis-induced myocardial dysfunction and mediates inflammation. Cytokine. (2016) 88:1–11. 10.1016/j.cyto.2016.08.006 27525353

[38] Furusho M Dupree JL Nave KA Bansal R. Fibroblast growth factor receptor signaling in oligodendrocytes regulates myelin sheath thickness. J Neurosci. (2012) 32:6631–41. 10.1523/JNEUROSCI.6005-11.2012 22573685 PMC3367512

[39] Liu J Dietz K DeLoyht JM Pedre X Kelkar D Kaur J. Impaired adult myelination in the prefrontal cortex of socially isolated mice. Nat Neurosci. (2012) 15:1621–3. 10.1038/nn.3263 23143512 PMC3729624

[40] Xiong X Gu L Zhang H Xu B Zhu S Zhao H. The protective effects of T cell deficiency against brain injury are ischemic model-dependent in rats. Neurochem Int. (2013) 62:265–70. 10.1016/j.neuint.2012.11.016 23228347 PMC3581747

[41] Joo IS Hwang DH Seok JI Shin SK Kim SU. Oral administration of memantine prolongs survival in a transgenic mouse model of amyotrophic lateral sclerosis. J Clin Neurol. (2007) 3:181–6. 10.3988/jcn.2007.3.4.181 19513129 PMC2686946

[42] Crawley JN. Behavioral phenotyping strategies for mutant mice. Neuron. (2008) 57:809–18. 10.1016/j.neuron.2008.03.001 18367082

[43] Kim SM Kim H Lee JS Park KS Jeon GS Shon J. Intermittent hypoxia can aggravate motor neuronal loss and cognitive dysfunction in ALS mice. PLoS ONE. (2013) 8:e81808. 10.1371/journal.pone.0081808 24303073 PMC3841127

[44] Kim HM Shin HY Jeong HJ An HJ Kim NS Chae HJ. Reduced IL-2 but elevated IL-4, IL-6, and IgE serum levels in patients with cerebral infarction during the acute stage. Journal of molecular neuroscience: MN. (2000) 14:191–6. 10.1385/JMN:14:3:191 10984195

[45] Okazaki K Nishida A Kato M Kozawa K Uga N Kimura H. Elevation of cytokine concentrations in asphyxiated neonates. Biol Neonate. (2006) 89:183–9. 10.1159/000089180 16244469

[46] Roka A Beko G Halasz J Toldi G Lakatos P Azzopardi D. Changes in serum cytokine and cortisol levels in normothermic and hypothermic term neonates after perinatal asphyxia. Inflamm Res. (2013) 62:81–7. 10.1007/s00011-012-0554-3 22986466

[47] Hu X Leak RK Shi Y Suenaga J Gao Y Zheng P. Microglial and macrophage polarization-new prospects for brain repair. Nat Rev Neurol. (2015) 11:56–64. 10.1038/nrneurol.2014.207 25385337 PMC4395497

[48] Hanisch UK Kettenmann H. Microglia: active sensor and versatile effector cells in the normal and pathologic brain. Nat Neurosci. (2007) 10:1387–94. 10.1038/nn1997 17965659

[49] Gadani SP Cronk JC Norris GT Kipnis J. IL-4 in the brain: a cytokine to remember. J Immunol. (2012) 189:4213–9. 10.4049/jimmunol.1202246 23087426 PMC3481177

[50] Derecki NC Cardani AN Yang CH Quinnies KM Crihfield A Lynch KR. Regulation of learning and memory by meningeal immunity: a key role for IL-4. J Exp Med. (2010) 207:1067–80. 10.1084/jem.20091419 20439540 PMC2867291

[51] McInnes A Rennick DM. Interleukin 4 induces cultured monocytes/macrophages to form giant multinucleated cells. J Exp Med. (1988) 167:598–611. 10.1084/jem.167.2.598 3258008 PMC2188835

[52] Nelms K Keegan AD Zamorano J Ryan JJ Paul WE. The IL-4 receptor: signaling mechanisms and biologic functions. Annual Rev Immunol. (1999) 17:701–38. 10.1146/annurev.immunol.17.1.701 10358772

