# Supplementary material for: Intranasal IL-4 Administration Alleviates Functional Deficits of Periventricular Leukomalacia in Neonatal Mice
Source: Front Neurol. 2020 Sep 2;11:930. doi: 10.3389/fneur.2020.00930 (PMC7492203; doi:10.3389/fneur.2020.00930)
Supplement: Supplementary file 1 [file Data_Sheet_1.PDF]

Antibodies	Corp.	Cat.	Diluted
MBP	abcam	ab209328	1:100
phosphor-IL4R	Invitrogen	PA5-38614	1:200
Iba-1	abcam	ab178846	1:1000
iNOS	abcam	ab15323	1:1000
Arg-1	abcam	ab60176	1:1000
TNF-a	abcam	ab8348	1:1000

Table S1: The detail parameters of primary antibodies in Western immunoblotting.

Gene	Species	FORWARD	REVERSE
TNF-a	Mouse	ATGTCTCAGCCTCTTCTCATTC	GCTTGTCACTCGAATTTTGAGA
Arg1	Mouse	CATATCTGCCAAAGACATCGTG	GACATCAAAGCTCAGGTGAATC
iNOS	Mouse	GTTTACCATGAGGCTGAAATCC	CCTCTTGTCTTTGACCCAGTAG
MBP	Mouse	CCCACTTGATCCGCCTCTTT	GCTGTGGGGTCTTCTTGGAT
IL-4R	Mouse	TGCCCTTATTTACTTTCGG	ACCCAGTCACCTCCTTTG
NG2	Mouse	CTTCCAGTTGAGCATGTCTGAT	TCACGATCGGAAATAACCTGAA
GAPDH	Mouse	GTGAAGGTCGGTGTGAACGG	GTTTCCCGTTGATGACCAG
Mag	mouse	GAGGATGATGGGGAATACTGG	TGTGACTCCAGAAGGATTATGG

Table S2: The detail primer sequences in the Real-time PCR.
