# Supplementary material for: Intranasal IL-4 Administration Alleviates Functional Deficits of Periventricular Leukomalacia in Neonatal Mice
Source: Front Neurol. 2020 Sep 2;11:930. doi: 10.3389/fneur.2020.00930 (PMC7492203; doi:10.3389/fneur.2020.00930)
Supplement: Supplementary file 2 [file Data_Sheet_2.pdf]

The negative control of protein in manuscript.

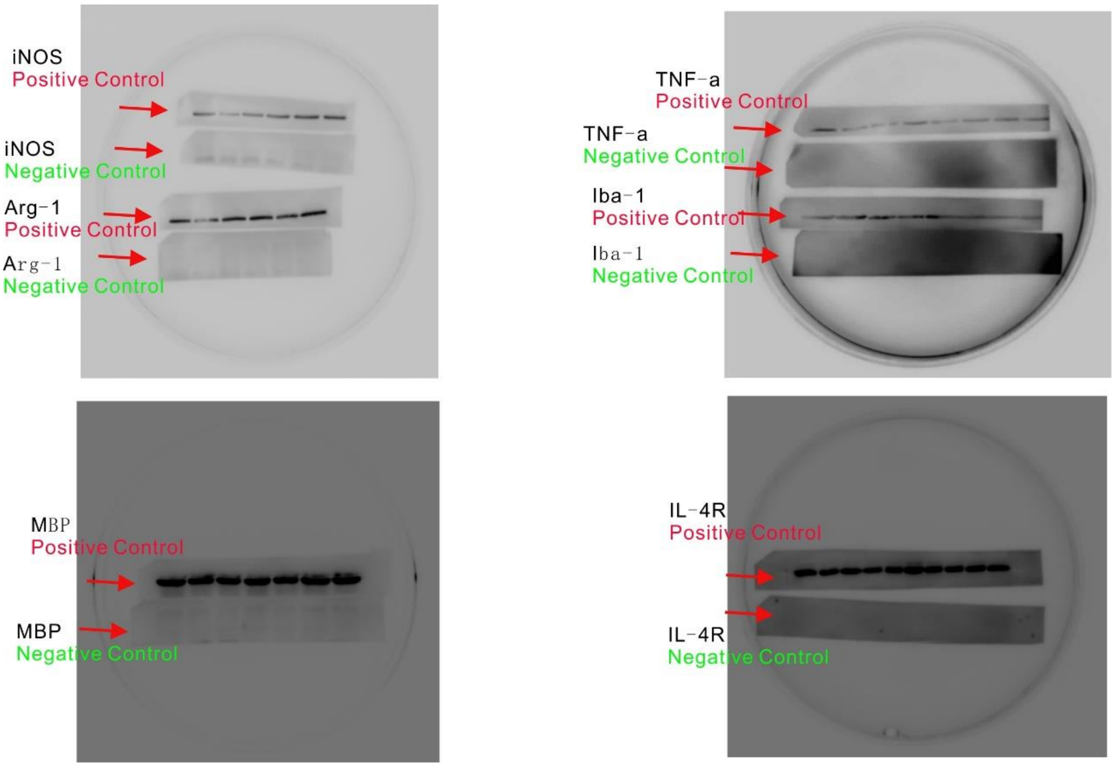

Figure S1. Western immunoblotting of protein in corpus callosum. The top panel is expression level of positive control, the bottom panel is negative control. Proteins were transferred onto PVDF membranes, and then positive control were incubated with different primary antibodies overnight, but negative control incubated with PBST, at 4°C. Then, all PVDF membranes were incubated appropriate secondary antibodies for 2 h at room temperature. ECL kit were used to detect protein expression.

The dose-response graph of IL-4 administration

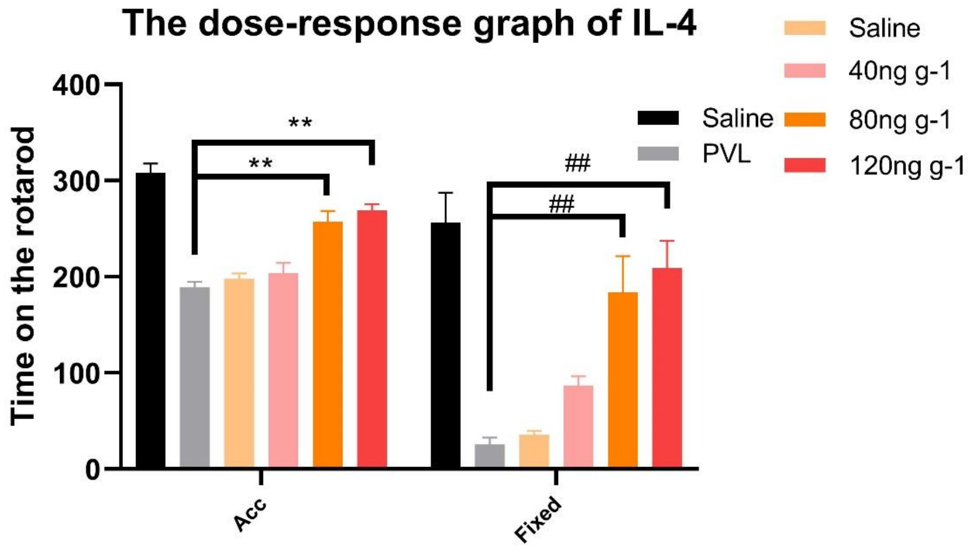

Figure S2. Rotarod test of mice exposed to RA, SAH, and SAH+IL-4 group, which subjected to the administration level of IL-4 for a total of 0, 40, 80, 120ng g-1 intranasally at P30. The functional differences were significantly detected at the level of 80, and 120ng g-1, but not at the level of 0, and 40 ng g-1.
